# The concepts of muscle activity generation driven by upper limb kinematics

**DOI:** 10.1186/s12938-023-01116-9

**Published:** 2023-06-24

**Authors:** Marie D. Schmidt, Tobias Glasmachers, Ioannis Iossifidis

**Affiliations:** 1 Faculty of Electrical Engineering and Information Technology, Ruhr-University Bochum, Bochum, Germany; 2 Institute of Computer Science, University of Applied Science Ruhr West, Mülheim an der Ruhr, Germany; 3 Faculty of Computer Science, Ruhr-University Bochum, Bochum, Germany

**Keywords:** Electromyography (EMG), Inertial measurement unit (IMU), Neural networks, Muscle activity, Motion parameters, Voluntary movement, Artificial generated signal, Generative model, Transfer learning

## Abstract

**Background:**

The underlying motivation of this work is to demonstrate that artificial muscle activity of known and unknown motion can be generated based on motion parameters, such as angular position, acceleration, and velocity of each joint (or the end-effector instead), which are similarly represented in our brains. This model is motivated by the known motion planning process in the central nervous system. That process incorporates the current body state from sensory systems and previous experiences, which might be represented as pre-learned inverse dynamics that generate associated muscle activity.

**Methods:**

We develop a novel approach utilizing recurrent neural networks that are able to predict muscle activity of the upper limbs associated with complex 3D human arm motions. Therefore, motion parameters such as joint angle, velocity, acceleration, hand position, and orientation, serve as input for the models. In addition, these models are trained on multiple subjects (*n*=5 including , 3 male in the age of 26±2 years) and thus can generalize across individuals. In particular, we distinguish between a general model that has been trained on several subjects, a subject-specific model, and a specific fine-tuned model using a transfer learning approach to adapt the model to a new subject. Estimators such as mean square error MSE, correlation coefficient r, and coefficient of determination *R*^2^ are used to evaluate the goodness of fit. We additionally assess performance by developing a new score called the zero-line score. The present approach was compared with multiple other architectures.

**Results:**

The presented approach predicts the muscle activity for previously through different subjects with remarkable high precision and generalizing nicely for new motions that have not been trained before. In an exhausting comparison, our recurrent network outperformed all other architectures. In addition, the high inter-subject variation of the recorded muscle activity was successfully handled using a transfer learning approach, resulting in a good fit for the muscle activity for a new subject.

**Conclusions:**

The ability of this approach to efficiently predict muscle activity contributes to the fundamental understanding of motion control. Furthermore, this approach has great potential for use in rehabilitation contexts, both as a therapeutic approach and as an assistive device. The predicted muscle activity can be utilized to guide functional electrical stimulation, allowing specific muscles to be targeted and potentially improving overall rehabilitation outcomes.

## Background

Human motion execution is the product of muscle contraction caused by muscle activation which, in turn, results from upstream motion planning of the motor cortical areas. Motion is defined as a change in position over time, and this can be described by parameters, such as time, direction, and velocity. Early motor control theories, such as Pavlov’s view of movements as combinations of reflexes and Sherrington’s proposal of reciprocal innervation to ensure coordinated movements, influenced the field [1, 2]. However, unlike Pavlov, Sherrington states that movements are generated by modulation of parameters of reflexes [2]. Bernstein formulates the equivalence problem, which highlights the redundancy in human motion where there are often more elemental variables than constraints associated with the movement [3]. For instance, when reaching a target in three-dimensional space, the number of arm joint rotations is typically more than three [4]. Bernstein’s theory suggests that the nervous system must choose a specific solution for each movement, taking into account the complex interplay between the nervous system, the musculoskeletal system, and the environment [3]. He proposes the existence of muscle synergy to simplify the control of multiple degrees of freedom [3, 5], which is compatible with Latash principle of abundance [4]. In the 1980s, Georgopoulos and colleagues found a correlation between the movement direction of the hand and the motor cortical activity [6, 7]. Moreover, speed and, with a less prominent effect, acceleration and position are continuously represented in motor cortical activity during reaching [8, 9]. There is some controversy about whether the motor cortex represents so-called high-level features of the hand as described above (direction, speed, and acceleration) or low-level features for muscle groups, such as muscle activity and force [10–12]. Churchland and colleagues developed a dynamical system approach to better understand the neural activity in the motor cortex [13]. Furthermore, the motor cortex might be explained by utilizing a recurrent neural network (RNN) [14–16] which in itself exhibits dynamical behavior. These models show that preparatory activity sets initial conditions that unfold predictably to control muscles during reaching. We might assume that the preparatory activity draws on pre-learned inverse dynamics that generate the associated muscle activity with measurable angular position, velocity, and acceleration for each joint. For further clarification, the term *“muscle activity”* refers to the neuronal signal at the muscle membrane that can be measured by surface electromyography (EMG), as in other works [17–20].

Building on this thesis, we demonstrate that muscle activity can be generated artificially for known and unknown motion based on high-level motion features for each joint (or for the hand instead), which is similarly represented in our brain [6–9]. For this, we develop a recurrent neural network with long–short term dependencies in a supervised learning session with motion parameters such as angular position, velocity, and acceleration of the arm. Previously trained motion can be generated with a remarkable precision, while new motions that are not previously trained reach a high precision in most cases. It has to be clarified that we do not aim to represent specific cortical activity but to show that muscle activity measured directly on the surface of the arm can be predicted based on motion parameters.

This concept is based on the assumption that motion parameters, such as angular motion, velocity, and acceleration, have a context to muscle activity. This assumption is in line with Georgopoulos and colleagues’ findings regarding the correlation between the movement direction of the hand and the motor cortical activity [6, 7]. This means that the specific parameters of a movement are encoded in the activity of the motor cortex, which then influences the activity of the muscles involved in that movement. Overall, this concept emphasizes the close relationship between motor control and the parameters of movement, and suggests that the two are intimately linked. Furthermore, it has been shown that the proprioceptive system which provides information about kinematic parameters, such as joint position, movement, and load [21] is crucial for adaption in reaching motion. The information of the proprioceptive system is transmitted to the central nervous system and integrated with other sensory systems like the visual system to generate an overall representation of the current body-state. Furthermore, the local loop in the spinal cord also has a direct influence on the generation of motion, especially on the time component where local parameters can be altered. This could be achieved by integrating additional hidden variables; however, this is part of future work and not incorporated in the present model.

There are several studies dealing with the inference of muscle activity based on measured motion parameters. According to the existing literature, two different approaches can be used: an analytical or a machine learning method. The analytical method is based on a biomechanical model that utilizes the given trajectory to compute muscle activity [22, 23], while the machine learning approach often involves artificial neural networks that directly learn the association between muscle and motion based on the data rather than on predefined programmed instructions. In both cases, these models often use joint angles, primarily those of the shoulder and elbow, or hand trajectories, as input parameters to predict the corresponding muscle activity [18, 23–26]. The task design, and thus the movements performed and muscle activity predicted, vary greatly throughout the existing literature: from simple one-dimensional motion to rare three-dimensional motions. Typically, $8-12$ muscles are recorded from $5-9$ participants. In the following, we take a closer look at the different machine learning-based approaches, where a feedforward network (FNN) is commonly used [17, 18, 26]. The performance is typically evaluated using the mean squared error (MSE), root-MSE (RMSE), normalized-RMSE (NRMSE), correlation coefficient r [27] or less commonly the coefficient of determination *R*^2^ and the variance accounted for (VAF), see "Evaluation method" section. We present our results with several of these methods to facilitate comparability. The comparison of different probabilistic methods by Johnsen and Fuglevand concludes that the dynamic neural network, which is a FNN with some time-delayed inputs, achieves good results with an average accuracy of r^2^= 0.40 for random three-dimensional motions [19]. Several other studies reported also good results employing FNN. Rittenhouse et al. achieved an average accuracy of *r*^2^= 0.66 with an FNN for a press-up motion [26]. Tibold and Fuglevand also used an FNN and achieved a *R*^2^=0.43 for loaded and unloaded random three-dimensional motion [17]. Alternatively, a probability-based prediction achieves the best accuracy with a VAF score of $69\%$ and an RMSE of up to 0.037 for the teres major muscle for two-dimensional random movements [20].

Here, we developed a specific recurrent network which is the most appropriate model for generating time series data due to its ability to recognize time dependencies in the motion sequence, and thus is the canonical candidate to approach the relation between motion and muscle activity. More precisely, we formed a Long–Short-Term Memory (LSTM) network, which is a type of recurrent neural network that allows for stimulation from earlier input remaining as hidden states to influence predictions at the current time step. This enables recurrent networks to exploit a dynamically contextual window which then can be utilized for time dependency of muscle activity. RNNs are not only suitable for this special case, and they are also generally suitable for physiological applications [28]. In addition to the recurrent network, we consider and evaluate other neural network architectures. A vanilla feedforward neural network (FNN) is a base model which allows the signal to only travel in one direction from input to output. Furthermore, a convolutional neural network (CNN) is introduced, which extracts spatial and temporal dependencies of the time series by applying different sized kernels and filters using the whole movement (and not just one point in time) as input. Thereby, the motion data as a whole are mapped to the muscle activity. Each neuronal network predicts the activity of all muscle simultaneously taking the synergistic nature of agonist and antagonist muscles into account. The performance of a model is presented by the mean square error (MSE) as the loss function of the neural network as well as the correlation coefficient *r* (often used squared $r^2$) and the coefficient of determination $R^2$. We further introduce a new better-suited measure, the *zero-line score*, that adaptively rescales the loss function of the muscle activity and compares the generated signal to the overall range of the muscle activity and thus identifies similarities between both signals. Unlike the similar defined $R^2$, the zero-line score uses the zero-line rather than the signals mean as comparison.

The generalization properties of the architecture are mandatory for all kinds off application along with the question of how transferable the feature is. We aim for a model which can further extrapolate across multiple subjects as well as for new motions. To provide a measure for generalization, we evaluate different disparate motions and transfer learning across all feature combinations across multiple subjects. The muscle activity, recorded by EMG, is known as a high inter-subject variable due to varying physiological factors, slightly different electrode placement, and other skin conditions [29–34]. Therefore, we evaluate models that have been trained on different individuals: we distinguish between a general model trained on multiple subjects but exclude the data from the subject that is referred to test these models, a subject-specific model that is trained entirely on this one subject, and a specific, fine-tuned model that uses the general model as a basis and is then adapted to the specific subject afterwards.

To evaluate the true generalization properties of our approach, we go beyond generating muscle activity based on already known motions and predict new, unseen motions covering a huge range of motions. Finally, we fall back on the input parameters we have chosen: angular position, velocity, and acceleration. We further evaluate and quantify the importance of all these motion parameters with respect to their contribution to generate artificial muscle activity or whether the redundancy of velocity and acceleration due to their reproducibility by deriving the position can be observed as well. In addition, we check if the position and orientation for the hand alone, also known as end-effector (EEF), are sufficient to drive the muscle activity of the upstream arm joints/segments, which can be described by inverse kinematics based on the EEF.

The objective of this study is to showcase the feasibility of inducing muscle activity via a kinematic representation. Previous research provides evidence that the brain’s neural activity encodes kinematic representation to some extent [6–9]. In this study, we substituted the neural representation of kinematics with actual measured kinematic parameters to generate a movement command. As a result, we developed a powerful generative model capable of generating muscle activity for new subjects and movements based on motion parameters similarly represented in the brain [6–9]. This highlights the close relationship between kinematic representations and muscle activity during motor control. This does not only contribute to a deeper understanding of motion control but has also practical applications in human–machine interaction and rehabilitation. In particular, this model addresses the crucial issue of subject-specific adjustments in myoelectrical controlled systems, such as prostheses and exoskeletons. These systems rely on the classification of residual signals to support or execute desired movements, and deep learning models require large data sets to achieve the performance and robustness necessary for real-world applications. The proposed muscle activity generation approach can generate the necessary subject-specific data to improve the performance and robustness of these systems. This generative approach has the potential to greatly benefit prosthetic users by allowing them to improve upon their existing movements with high robustness and accuracy. Additionally, it can go beyond the limitations of a set of pre-programmed motions by exploring new, generative approaches that may produce less accurate but more diverse and creative movements. Furthermore, in rehabilitation, the generated muscle activity can serve as a building block for targeted functional electric stimulation (FES) of paralyzed limbs to support movement.

## Methods

In this section, the entire process from data acquisition to pre- and post-processing to the construction of the different neural network architectures and their hyperparameter tuning is outlined.

### Experimental protocol

**Fig. 1:**
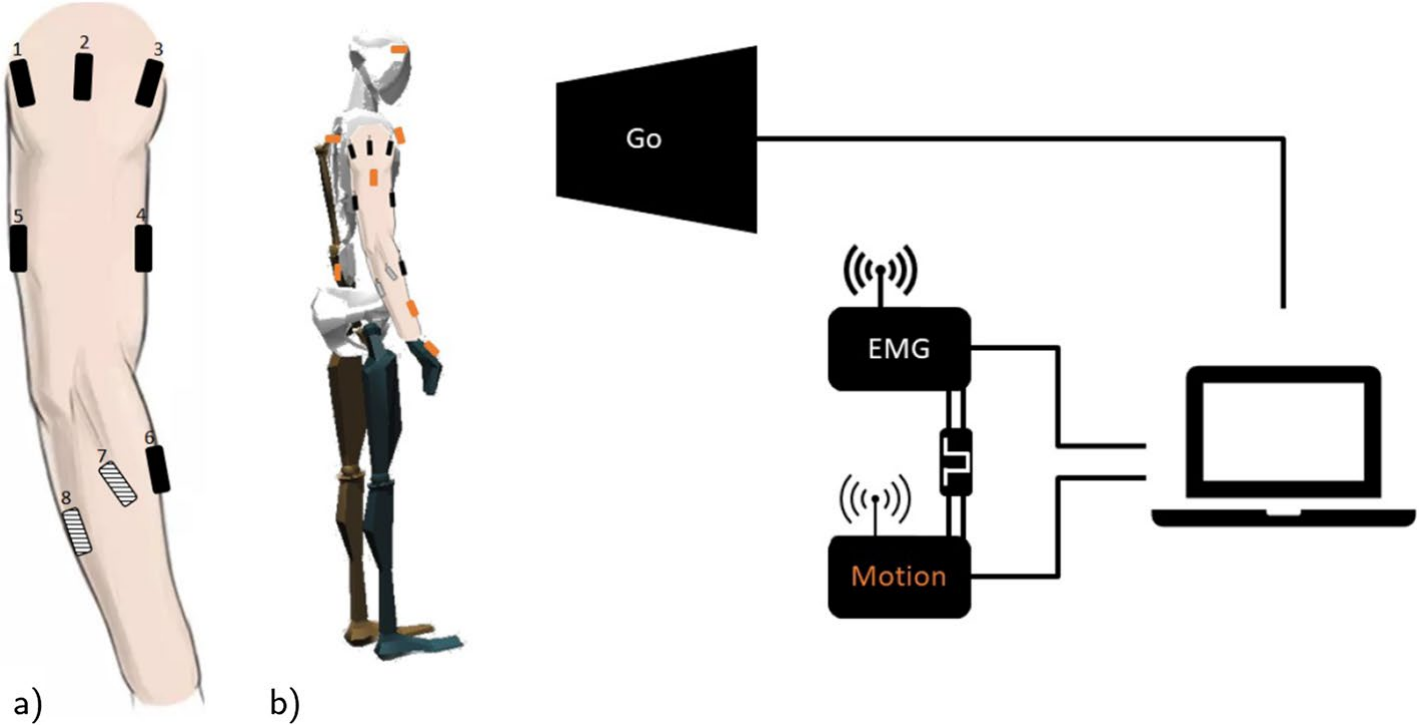
**a** Overview of the electrode (rectangles) placement on the right upper limb (deltoid posterior (1), lateral (2) and anterior (3), biceps short head (4), triceps brachii lateral head (5), extensor carpi radialis longus (6), pronator teres (7), and flexor carpi ulnaris (8) with the last two shaded), modified from [37]. **b** Overview of the test setup with the subject standing in front of the screen and the following measurement device, Delsys Trigno EMG System and Xsens motion capture system (motion sensor in orange) connected to the the trigger module and the laptop

**Fig. 2:**
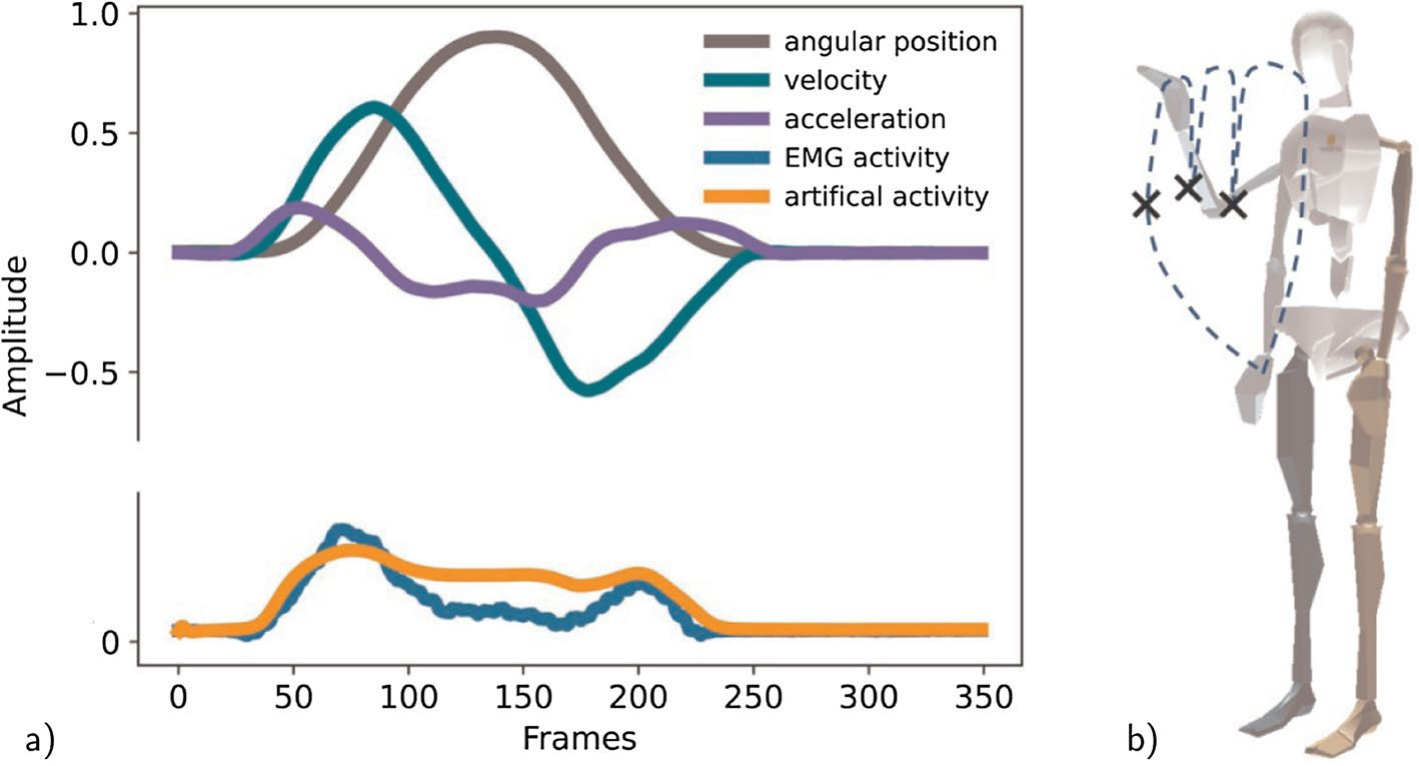
**a** Motion trajectory (in terms of angular position, velocity, and acceleration) and muscle biceps signal as well as the artificially generated signal for flexion and extension of the elbow. **b** Visualization of the new motion, pointing to three points in space (the three points are indicated by the black crosses and trajectory indicated by the dashed line)

In total, five healthy subjects (2 female, 3 male in the age of $26~ \pm 2$ years) participated in the experiment. They all have given their informed written consent to the study. The study involving human subjects was reviewed and approved by the Ethics Committee of the Ruhr-University Bochum. All methods were performed in accordance with the relevant guidelines and regulations. Each subject performed 20 tasks with 18 repetitions of each isotonic movement resulting in 360 motion sequences per subject. An isotonic movement is caused by a muscular contraction that leads to a change in muscle length and thereby causes a motion at the corresponding joint. The movements can be categorized into three groups: simple motion, combined, and complex motion (Table 1). The simple motions include shoulder flexion to 90 degrees, shoulder extension, shoulder abduction to 90 degrees, elbow flexion, elbow flexion with a supinated forearm, wrist flexion, wrist extension, and wrist pronation. Unless specified differently, all simple motions are performed to their maximum flexion and extension, respectively, as long as it is comfortable, accepting a small inter-subject variability; there is no external target that must be reached. The combined movements are composed of shoulder abduction elbow flexion, shoulder flexion elbow flexion, and shoulder abduction wrist extension. The complex movements try to mimic everyday activity’s as breaststroke (extend the arm in front of the body center, pronate the forearm and perform a horizontal extension), relay handover (shoulder extension and pronation of the forearm, so that the relay can be passed), reading a clock (raising the forearm in front of the body and pronation of the forearm, so that the time can be read from the wristwatch), diagonal reach (using the right hand to lightly touch the left arm at different heights), waving gestures, drawing a circle in the air (in front of the body), and pointing to three points in space (Figs. 1 and 2). Each movement begins and ends in a rest position with the arm relaxed and hanging parallel to the side of the body. The subjects are instructed to perform a controlled movement and must not to use gravity to return to the rest position, e.g., in shoulder abduction, the arm is first raised to a certain degree, followed by the downward movement, which should be performed in a controlled active manner and not passively use gravity.

**Table 1:** List of all exercises considered for this project, each task is repeated 18 times

Simple motion	Shoulder flexion
	Shoulder flexion (mix)
	Shoulder extension
	Shoulder abduction
	Shoulder abduction (mix)
	Elbow flexion
	Elbow flexion (mix)
	Elbow flexion with a supinated forearm
	Wrist flexion
	Wrist extension
	Wrist pronation
Combined motion	Shoulder abduction with simultaneous elbow flexion
	Shoulder flexion with simultaneous elbow flexion
	Shoulder abduction with simultaneous wrist extension
Complex motion	Breaststroke
	Relay handover
	Reading a clock
	Diagonal reach
	Waving gestures
	Drawing a circle
	Pointing to three points in space*

All simple and complex movements were performed to a natural joint maximum. Shoulder flexion and abduction were performed only to 90 degrees. Movements with the addition of (mix) are performed with arbitrarily changing endpoint, i.e. the subjects are allowed to stop their movement before their natural joint maximum. *This movement is just performed by the test subject

The muscle activity of these movements is recorded at 2222 Hz using the Trigno Wireless EMG System (Delsys Inc., Boston, MA, USA) with two Quadro electrodes. The skin preparation and placement of electrodes were performed according to the recommendation of the SENIAM manuscript [35]. Eight EMG electrodes were placed on the upper right arm on the following muscles: deltoid anterior, medial, and posterior, biceps short head, triceps brachii lateral head, pronator teres, flexor carpi radialis, and extensor carpi ulnaris (Fig. 1). The targeted muscles were selected according to an antagonistic pattern and extended over several joints, thus covering multiple degrees of freedom. Note that the musculoskeletal system has a certain redundancy and multiple muscles contribute to one movement. We have selected only a representative selection of these muscles. The EMG system is start and stop synchronized with the motion tracking from Xsens Motion Capture via the Delsys trigger box. The EMG signal is processed with a root mean square and sampled at a frequency of 60 Hz matching the timestamps of the Xsens Motion Capture system. For this, we place a window of 200 ms around each Xsens timestamp. Sequentially, we select all timestamps of the EMG signal that are within this window and calculate their root mean square. In this way, the EMG signal is synchronized with the Xsens data while being simultaneously smoothed and downsampled. The Xsens Motion Capture system (Xsens Technologies B.V., P.O. Box 559, 7500 AN Enschede, Netherlands) uses the upper body configuration including 11 sensors covering both arms and the torso, i.e., wrist, forearm, upper arm, shoulder for each side, sternum, pelvis, and head. The application and advanced N-pose calibration of the sensors is performed according to their manual [36]. The Xsens Motion Capture samples with a rate of 60 Hz. During the visual validation of the motion capture performance, it was found that the abduction and adduction of the wrist is not very accurate and can deviate up to a maximum of 20 degrees, therefore, simple and combined motions that include wrist abduction and adduction were not included. This issue could be improved in the future with the incorporation of additional sensors.

During the experiment, the subjects stand in front of a screen showing the visual interface which provides the instructions always starting with the resting position for 4 s signaled by the ‘resting window’. Next, an instruction window pops up and refreshes every 7.5 s indicating the next repetition. During this time, the subject is asked to perform the described movement repeatedly. To avoid muscle fatigue, 60 s of rest is granted after each task.

### Data preprocessing

The EMG signal is baseline corrected, and outliers are determined by considering values with six standard deviation of the mean. The detected outliers are mostly related to cable movement and are fitted and subtracted by a spline. Afterwards, the EMG data is smoothed with a root mean square (1) over a window size of 200 ms and simultaneously downsampled to the synchronized Xsens frequency of 60 Hz. Further, for each subject individually, all EMG and motion data channels are normalized between 0 and 1 and $-1$ and 1, respectively. The normalization is necessary due to huge differences between the individual channels. Furthermore, the data are cut into motion sequences of 457 frames where each motion sequence represents one completed motion to eliminate inter-trial pauses. The onset and offset of motion sequences are dictated by instructed cues. By using a fixed window size, we are able to include longer trials that encompass both active and non-active states, thereby facilitating learning of both states. The Xsens motion capture system provides the hand position, orientation as well as angular position of each joint. The latter includes the angular position of the shoulder, elbow, and wrist reflecting shoulder abduction/adduction, shoulder extension/flexion, elbow extension/flexion, elbow rotation, wrist abduction/adduction, and wrist extension/flexion is extracted. The angular position is filtered by a third-order Savitzky–Golay filter [38] and then two times discretely differentiated to compute the angular velocity and acceleration using the forward difference operator $\Delta f:n \mapsto f (n+1)-f(n)$. The angular position, angular velocity, and angular acceleration of each joint serve as input data for the following models. In addition, the hand position, and orientation are used for the EEF-configuration and their corresponding velocity-, and acceleration for the EEF^+^-configuration.

1 $$\begin{aligned} \textrm{RMS} = \sqrt{\frac{1}{n}\sum _{i=1}^{n} x_i^2} \end{aligned}$$

### Neural network models

We investigate the relationship between motion input data and muscle activity that can be learned by a neural network to generate an artificial muscle activity. The models are trained on angular position, velocity, and acceleration for each joint to predict the corresponding muscle activity. In a matter of supervised learning, we compare the predicted muscle activity to the smoothed (1) recorded muscle activity. The recurrent neural network is compared to two other network types: a basic vanilla feedforward network and a more complex convolutional network. Besides the different architectures, the models are also evaluated on different training approaches. These approaches include training on different subjects’ data: distinguishing between a general model that has been trained on several subjects, a general model which was fine-tuned on subject-specific data, and an exclusively subject-specific model trained on the data of one subject (the last recorded subject) only. The architectures are evaluated by the general model trained and tested on multiple subjects ("Comparison of the network architectures" section). For the evaluation of the different training approaches, one subject is excluded from the training data set to, later on, test the model on unseen subject data (Results "Performance for new subjects" section). Therefore, the training set consists of 15 repetitions of $n-1$ motions (all motions except one), leaving 2 repetitions for $n-1$ motion for the training set and one repetition $n-1$ motion for the validation set. The excluded $n-1$ motion is later used to evaluate the performance to generate new motions (Results "Generalization property for new motion" section). Finally, we demonstrate with the Leave-One-Out (LOO) method that the results obtained above can be reproduced with a separate subject from all other subjects (see "Leave One Out method: determine variation between subjects" section). In the LOO method, one other subject is left out of each run such that the model can be evaluated based on this subject. After running across all subjects, the average value can be determined. We also removed two motions from the pool and used these as a new test data set for the new motion. These are a simple shoulder flexion and a complex relay handover motion. In "How many repetitions of a motion are required for learning?" section we analyze how many repetitions of a movement are required for learning.

The FNN is feed with a vector containing multiple motion sequences at ones, whereas the LSTM and CNN are feed with one motion sequence at a time. Each neuronal network predicts the activity of all muscle simultaneously taking the synergistic nature of agonist and antagonist muscles into account.

All networks are implemented with the Keras API [39]. The networks use an adaptive learning rate optimization algorithm called Adam [40] to change the learning rate and weights to reduce the loss. The loss is the prediction error of the neural network computed in our case by the mean squared error loss function (2). Through backpropagation, the loss is transferred from one layer to another and the weights are modified depending on the losses so that the loss is minimized. The rectified linear activation function (ReLu) (3) is used in the hidden layers which describe the transformation from input to output from a node. To prevent overfitting, an early stopping with a patience of 5 epochs and dropout layers (that can randomly set input units to zero) are implemented.

2 $$\begin{aligned} \textrm{MSE}&= \frac{1}{n}\sum _{i=1}^{n} (y_i-x_i)^2,\end{aligned}$$

3 $$\begin{aligned} f(x)&= \textrm{max}(x,0)= {\left\{ \begin{array}{ll} x &{} \textrm{if}\;\; x >0, \\ 0 &{} \textrm{otherwise}\, \end{array}\right. } \end{aligned}$$

#### Recurrent neural networks

In general, recurrent neural networks (RNN) are specialized in time dependencies due to their specific architecture that allows adding information of previous time steps to the current one. Theoretically, all recurrent networks are stateful, however, in Keras, this only applies within a batch. If an additional time sequence is separated into individual batches, the previous information is lost since the states are initialized at the first time step of a batch and reset to 0. Two possibilities are derived from this: either the batch contains the entire sequence of one movement, or the state must be regained during warm-up phase before the actual prediction of the current time step. The latter suggestion can also be used for an online prediction of the muscle activity. In the following, both approaches are applied beginning with the first approach, which trains and predicts the whole sequence at once.

**Fig. 3:**
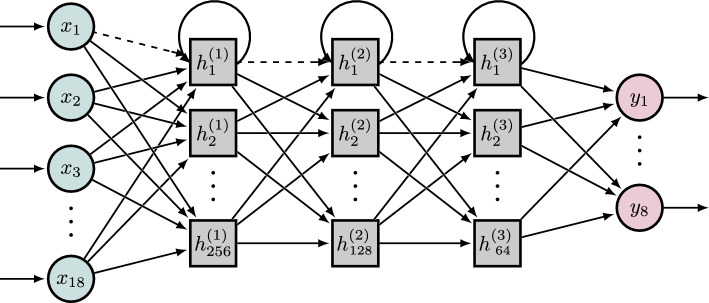
Recurrent neural network architecture with a linear motion input (green circle), three LSTM hidden (gray square), and linear muscle output (red circle) layer. The arrows that connect nodes $h_{1}^{(1)}$, $h_{1}^{(2)}$, and $h_{1}^{(3)}$ back to themselves are representative for all LSTM gray square nodes. The dashed connection indicated a dropout layer

The recurrent network in this work consists of an input dropout and three hidden Long Short-Term Memory (LSTM) [41] layers (256, 128, 64 nodes) and a TimeDistrubtion dense output (Fig. 3). The LSTM network is a type of RNN that overcomes the vanishing and exploding gradient problem of standard RNNs [41] by using gates to control the memorizing process. The model is trained with a vector of dimension 3: total number of sequences, time steps of each sequence, and number of features. In the LSTM layer, the return sequence and stateful parameter are enabled, which allows the LSTM layer to predict the whole sequence at once. To obtain a good result at the beginning of the sequence, the stateful parameter needs to be enabled. Thus, the memory states and hidden states of the LSTM layer are saved from the former sequence and used as a reasonable starting value for the next sequence. In our context, this works particularly well because every sequence starts and ends at the same resting position ensuring that each sequence has a very similar starting value range. However, the downside of this approach is a slower backpropagation due to the length of a whole sequence.

In the second approach, the recurrent network is trained with fractures of the motion sequence, called sub-sequences. Each sub-sequence only consists of a single data point in time, which has to be predicted, and a few prior time steps $i:20+i$ as a warm-up phase, solely used to restore the correct time-dependent states of the recurrent network. In addition, this approach benefits from two models, one for training using the sub-sequence and one for prediction using the whole sequence again allowing different batch sizes for training and testing. While it is more efficient to train with a higher batch size it is necessary to predict with a batch size of one for each time step. Both models, the RNN and the sub-sequences model RNNseq have a similar architecture starting with an input dropout followed by a LSTM layer(s) and a dense output layer. The stateful parameter in the LSTM layer is enabled in both networks. The return sequence parameter is not needed in this scenario because we are not training on whole sequences. In order to predict a whole sub-sequence in one, a new model needs to be defined using the same weights as for the previously trained model. The architecture only differs in the batch size parameter of one. The states will be rest after a full sequence prediction. This approach can also be used for online generation of muscle activity.

#### Feedforward network

**Fig. 4:**
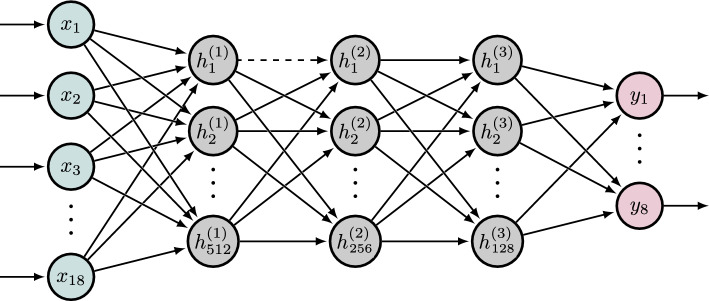
Feedforward neural network architecture with motion input, three hidden, and muscle activity output layer. The dashed connection between $h_{1}^{(1)}$ and $h_{1}^{(2)}$ indicates the dropout layer

The vanilla feedforward network (FNN) is one of the more basic networks with a simple forward pass of information. The architecture is composed of three fully connected hidden dense layers with 512, 256 and 128 nodes, and an 8-node output according to the number of predicted EMG channels (Fig. 4). The model is trained with a batch size of 128, i.e., the gradient is updated every 128th sample. In theory, it should be beneficial to have additional information on previous time steps to predict the current time step. Therefore, we develop a vanilla network that is also fed with sequence information (FNNseq) of additional previous time steps $n-i^2=(n-1,n-2,n-4,n-8,..)$, which are added to the feature input vector. This information of previous time steps can improve the prediction of the current step.

#### Convolutional neural network

**Fig. 5:**
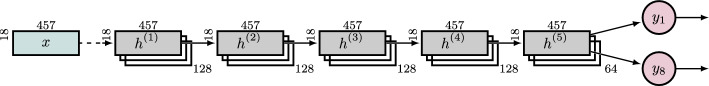
Convolutional neural network architecture with a motion input (green circle), three Conv1D hidden (gray square), and linear muscle output (red circle) layer. The dashed connection indicates a dropout layer

Besides the recurrent, the convolutional neural network (CNN) is also able to capture time dependencies through the application of relevant filters. The CNN works as a feature extractor that transforms the data into a form that is easier to process, with the intention not to lose relevant information necessary for a good prediction. This is done by the kernel and filter parameters in the convolutional layer. The CNN consists of an input dropout layer with a rate of 0.1 followed by 5 one-dimensional convolution layers with decreasing number of convolutions (128, 128, 128, 128, 64) and kernel size of (32, 8, 8, 4, 4) and a final output dense layer with 8 nodes (Fig. 5).

### Hyperparameter tuning

All models were hyperparameter tuned using optuna for optkeras with enabled pruning option on an evolutionary sampler [42]. Concerning a minimal validation error the following parameters were optimized: batch size, number of layers, number of nodes, the dropout rate, and number of filter and kernel size for the CNN.

### Evaluation method

To evaluate the similarity with the smoothed original muscle activity and compare between all artificially generated signals, the MSE (2) is computed and is also used as a loss function by the neural networks. For the MSE, a smaller value indicates a more similar signal. The over-representation of lower values caused by the rest state and several inactive muscle groups for most motions strongly influences the MSE, which in this case inherents lower values than expected. Note that the MSE of the whole original data-set is only 0.0044 while the signal is allowed to have values up to 1. Therefore, this metric is not ideal to compare the results of different task designs. Besides the MSE also the correlation coefficient r (4) is commonly used to evaluate the difference between the original and predicted signal [27]. The correlation coefficient describes the relationship or rather the connection between two signals, where 0 indicates a lack of correlation, 1 perfect correlation, and $-1$ a negative correlation. Usually r^2^ is used instead to restrict the value between 0 and 1. Various works [27] used the r^2^ to quantify their prediction. It is important to note that *r*^2^ is not only independent of the magnitude of the original data values, which is desired, but also only sensitive to relative changes between original and generated data. For instance, a predicted signal which is exactly half of the value of the original signal still has a correlation coefficient of 1. This is, however, not ideal to quantify the goodness of a fit. Both, the timing and in particular the amplitude of muscle activity are crucial for a realistic generation of artificial muscle activity. Therefore, the coefficient of determination *R*^2^ (5), which should not be confused with *r*^2^, is better suited to rate a fit in this context. *R*^2^ measures how well the predicted value matches the original value by considering the distance relative to the average of the original signal (5). The R^2^ reaches from any negative number to 1 indicating a perfect match.

Motivated by the weakness of the MSE to be sensitive to the over-representation of lower values and the *R*^2^ using a mean signal comparison, we introduce a new rating, the so-called zero-line score (6) which calculates a zero line signal comparison. With the mean square value of the original signal as a baseline, a score of 0 indicates an approximation as poor as the zero line signal itself, while a value of 100 signifies perfect alignment. The smaller the values of the original signal are, causing a closer resolution of the zero-line score, the more difficult it becomes to reach a high zero-line score for the artificially generated signal. This is due to the fact that the smallest possible zero-line score value decreases. For instance, the data used to evaluate the general model, the zero-line score can hypothetically attain a value of approximately $-5000$, that describes the error between the original signal and a hypothetical signal containing the respectively more distant limit 0 or 1 at each time step. The zero-line score is especially useful for capturing the error of muscle activity for multiple channels, which are of different magnitude. The main difference between the zero-line score and the *R*^2^ is the comparison of the MSE with the zero lines signal and the mean signal, respectively. The zero-line score is especially recommended for signals that have long periods of inactivity, i.e., signals close to zero. In the following, we will present all our results for the RNN with the MSE, *r*^2^, *R*^2^ and zero-line score to allow a comparison with other works.

4 $$\begin{aligned} \textrm{r}&= \frac{\sum \limits _{i=1}^{n} (x_i-{\overline{x}}) (y_i-{\overline{y}})}{\sqrt{\sum \limits _{i=1}^{n} (x_i-{\overline{x}})^2 \cdot \sum \limits _{i=1}^{n} (y_i-{\overline{y}})^2}}\,, \end{aligned}$$

5 $$R^{2} = 1 - \frac{{\sum\limits_{{i = 1}}^{n} {(y_{i} - x_{i} )^{2} } }}{{\sum\limits_{{i = 1}}^{n} {(y_{i} - \bar{y})^{2} } }}{\mkern 1mu} ,$$

6 $$Z_{s} = 100 \cdot \left( {1 - \frac{{\sum\limits_{{i = 1}}^{n} {(y_{i} - x_{i} )^{2} } }}{{\sum\limits_{{i = 1}}^{n} {(y_{i} - 0)^{2} } }}} \right).{\text{ }}$$

$$y_i$$: original data

$$x_i$$: predicted data

${\overline{y}},{\overline{x}}$: average of the original/predicted data

*n*: number of data

Note that the zero-line score *Z*_s_ as well as the *R*^2^ can easily reach values smaller than zero, e.g., when the predicted signal is higher than twice the original signal.

## Results

The results part is structured into several interrelated sections. First, we assess the overall suitability of various network architectures in generating synthetic muscle activity by comparing the prediction outcomes. Following this, we examine the model’s ability to generalize beyond the training data by predicting muscle activity for previously unseen subjects and motions. We then investigate the significance of input data in the model and explore how inter-subject variability affects the results. In addition, we determine the minimum number of repetitions required for learning and compare the effectiveness of subject-specific versus general models.

### Comparison of the network architectures

**Fig. 6:**
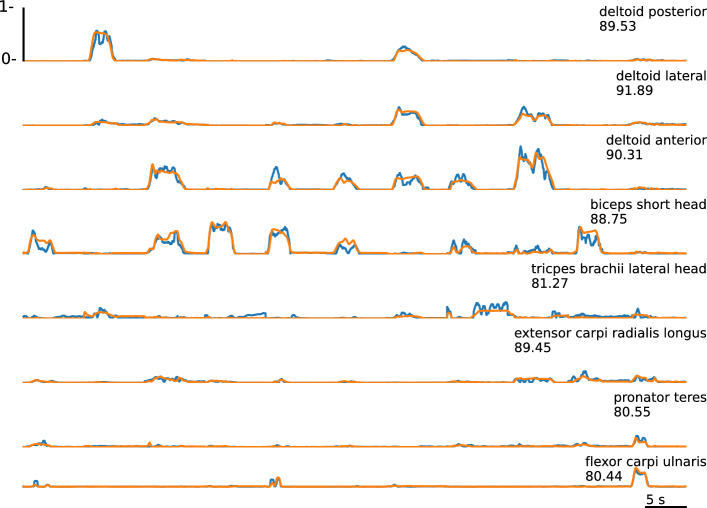
Muscle activity for all eight channels (blue) and the artificial generated muscle activity (orange). The artificial activity is generated on the general training approach using an RNN architecture, evaluated by the zero-line score. The muscle activity corresponds to the following motions: elbow flexion (mix), shoulder extension, shoulder flexion with simultaneous elbow flexion, elbow flexion with a supinated forearm, shoulder flexion with simultaneous elbow flexion, shoulder flexion with simultaneous elbow flexion, shoulder abduction, waving gestures, breaststroke, elbow flexion, and wrist flexion

Across all architectures, the artificial muscle activity is approximated reasonably close (Fig. 6). In the following, the architectures are evaluated based on a general model containing train and test data from multiple subjects. Thereby, the recurrent neural network outperforms the others with a zero-line score of 88.13 and an *R*^2^ value about 0.85 (Table 2). The sub-sequence based online recurrent neural network achieves the lowest score of 83.33. The scores for each channel reveal a higher accuracy for the second and third channels, which represent the deltoid muscle activity.

**Table 2:** Overview of the performance for all architectures (recurrent neural network (RNN), sub-sequenced input recurrent neural network (RNNseq), feedforward neural network (FNN), sub-sequenced input feedforward neural network (FNNseq), convolutional neural network (CNN)) and channels (electrodes 1-8) with the general approach (training and testing on multiple subjects) evaluated by the zero-line score Z_s_, mean square error MSE, the squared correlation coefficient *r*^2^, and the coefficient of determination R^2^

		1	2	3	4	5	6	7	8	Average
RNN	*Z*_s_	89.53	91.89	90.31	88.75	81.27	89.45	80.55	80.44	**88.13**
	MSE	0.0009	0.00131	0.00285	0.00203	0.00269	0.00066	0.00066	0.00081	0.00149
	*r*^2^	0.88156	0.89965	0.87858	0.84992	0.77071	0.87266	0.79592	0.80298	0.85597
	*R*^2^	0.87719	0.89916	0.87292	0.8474	0.75624	0.8695	0.77816	0.78616	0.84904
RNNseq	*Z*_s_	86.24	90.34	86.78	84.71	69.16	84.42	76.11	72.94	83.33
	MSE	0.00119	0.00156	0.00388	0.00275	0.00443	0.00098	0.00081	0.00113	0.00209
FNN	*Z*_s_	88.39	90.97	90.06	88.43	63.58	88.59	79.81	82.19	85.21
	MSE	0.001	0.00146	0.00292	0.00208	0.00523	0.00071	0.00068	0.00074	0.00185
FNNseq	*Z*_s_	88.6	91.67	89.74	88.57	75.35	86.73	78.21	81.71	86.81
	MSE	0.00102	0.00139	0.00311	0.00214	0.00356	0.00085	0.00077	0.00078	0.0017
CNN	*Z*_s_	88.49	91.24	89.78	88.25	71.94	86.59	79.89	81.0	86.18
	MSE	0.00099	0.00141	0.003	0.00212	0.00403	0.00084	0.00068	0.00079	0.00173

### Performance for new subjects

The next step is to evaluate how the model predicts new unseen subjects. For this purpose, the general model from the  "Comparison of the network architectures" section is first tested on a separate subject. In this setup, all architectures reach a significantly lower similarity between 30 and 40 (Table 3). This is not surprising due to inter-subject variance for further information see "Discussion" section.

**Fig. 7:**
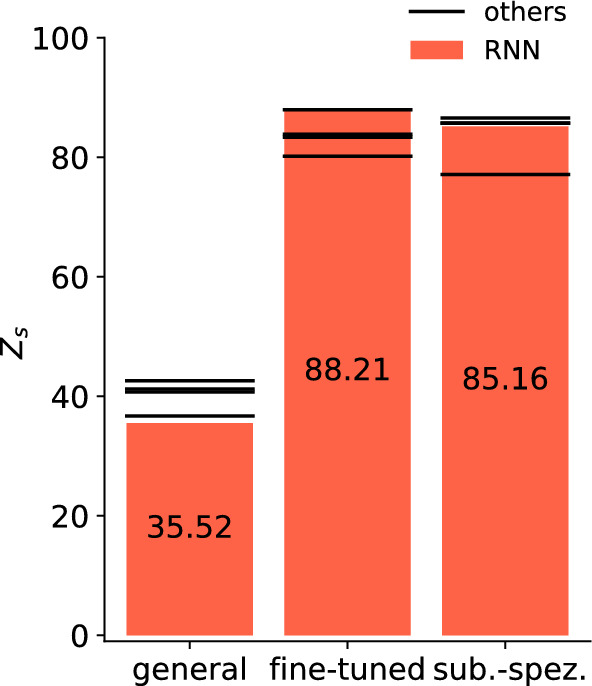
New subject performance for the RNN (orange bar) of the general, fine-tuned and subject-specific model. The other network architectures (RNNseq, FNN, FNNseq and CNN) are represented by the black lines

To improve the performance, we apply a subject-specific fine-tuning on the general model using transfer learning with a weight initialization strategy [43]. Thereby, the weights of the original general model are used as a pre-training status which is then updated on the subject-specific data. This approach leads to a significant increase in performance for all architectures and rer values to the multiple-subject setting described in the"Comparison of the network architectures" section with a slightly higher maximum of 88.21 for the zero-line score and around 0.86 for the *R*^2^ value for the RNN (Table 3, Fig. 7). For comparison, we further train a model purely on one subject’s data, i.e. the data from the previously separate subject, resulting in a subject-specific model. This model again has a high performance of *Z*_s_ > 80 across most architectures (Table 3, Fig. 7) but remains behind the fine-tuned model. We further comp general approach with the subject-specific approach in "Subject-specific or general model" section.

**Table 3:** All architectures (recurrent neural network (RNN), sub-sequenced input recurrent neural network (RNNseq), feedforward neural network (FNN), sub-sequenced input feedforward neural network (FNNseq), convolutional neural network (CNN)) are tested on the same test data set of the separate subject and evaluated by the zero-line score *Z*_s_, mean square error MSE, the squared correlation coefficient *r*^2^, and the coefficient of determination *R*^2^

		General	Fine-tuned	Subject-specific
RNN	*Z*_s_	35.52	**88.21**	85.16
	MSE	0.00747	0.00137	0.00172
	*r*^2^	0.3338	0.86077	0.83648
	*R*^2^	0.21422	0.85634	0.81915
RNNseq	*Z*_s_	41.23	83.36	77.12
	MSE	0.00681	0.00193	0.00265
FNN	*Z*_s_	36.68	83.83	85.74
	MSE	0.00733	0.00187	0.00165
FNNseq	*Z*_s_	40.71	87.95	85.67
	MSE	0.00714	0.00145	0.00173
CNN	*Z*_s_	42.61	80.18	86.56
	MSE	0.00665	0.0023	0.00153

The results are presented as an average over all channels, with the bold number indicating the highest score

### Generalization property for new motion

**Fig. 8:**
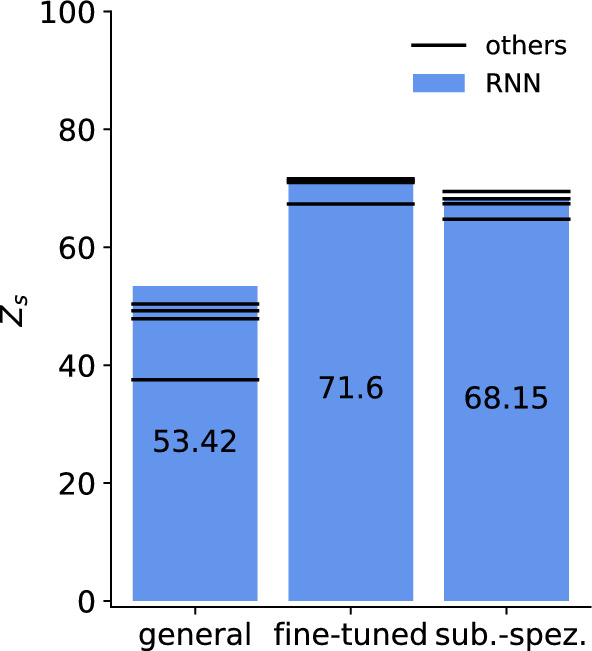
New motion performance for the RNN (blue bar) of the general, fine-tuned and subject-specific model. The other network architectures (RNNseq, FNN, FNNseq and CNN) are represented by the black lines

**Fig. 9:**
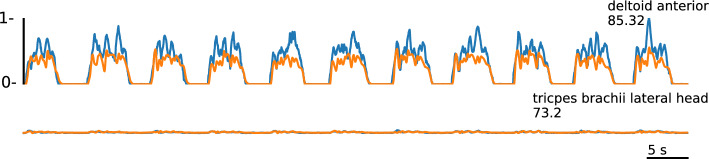
Muscle activity (blue) from a new motion (pointing into 3 points in space) and the artificial generated muscle activity (orange). The artificial activity is generated after fine-tuning using an RNN architecture

Thus far, we have shown that we can predict muscle activity with different architectures and also generate subject-specific muscle activities. The natural consequence of this is to go beyond can we generate new motion that has not been learned before. For this, all models are tested on a new motion not seen before, pointing to 3 points in space (Fig. 2b) for the separated subject introduced in "Performance for new subjects" section. We want to emphasize that we evaluate a new type of movement only for the separated subject. The new motion is not performed by any of the other subjects. Overall, the fine-tuned models reach a consistently higher accuracy than the general and subject-specific model, led by the RNN and CNN with 72 (Table 4, Fig. 8). Previously, the *R*^2^ reached similar high rankings as the zero-line score see Table 2 and 3, but for the new motion, the R^2^ remain comparatively low for the fine-tuned model at 0.36 (Table 4). In all cases, the fine-tuned models also outperform the subject-specific model for a more detailed comparison see "Subject-specific or general model" section. Note that the *R*^2^ can be negative if the numerator MSE which is proportional to the MSE is greater than the sum of the squared differences between $y_i$ and ${\bar{y}}$ (5) as it is the case for general model predicting a new motion (Table 4). EMG channels that tend to have a large contribution to the motion and, therefore, have increased amplitudes achieve higher accuracy *R*^2^=0.67401 and *Z*_s_= 85.32 in generating new motions compared to channels with reduced activity *R*^2^=0.39 and *Z*_s_= 73.2 (Fig. 9).

**Table 4:** All architectures (recurrent neural network (RNN), sub-sequenced input recurrent neural network (RNNseq), feedforward neural network (FNN), sub-sequenced input feedforward neural network (FNNseq), convolutional neural network (CNN)) are tested on a new motion from the separate subject and evaluated by the zero-line score *Z*_s_, mean square error MSE, the squared correlation coefficient r^2^, and the coefficient of determination *R*^2^

		General	Fine-tuned	Subject-specific
RNN	*Z*_s_	53.42	**71.6**	68.15
	MSE	0.01286	0.00784	0.00879
	*r*^2^	0.64099	0.71044	0.7245
	*R*^2^	– 0.05092	0.35933	0.28147
RNNseq	*Z*_s_	49.22	71.27	67.39
	MSE	0.01401	0.00793	0.009
FNN	*Z*_s_	50.39	67.33	64.76
	MSE	0.01369	0.00902	0.00973
FNNseq	*Z*_s_	37.51	70.99	69.47
	MSE	0.01786	0.00829	0.00873
CNN	*Z*_s_	47.87	**71.63**	68.22
	MSE	0.01439	0.00783	0.00877

The results are presented as an average over all channels, with the bold numbers indicating the highest score

### Input parameter validation

**Fig. 10:**
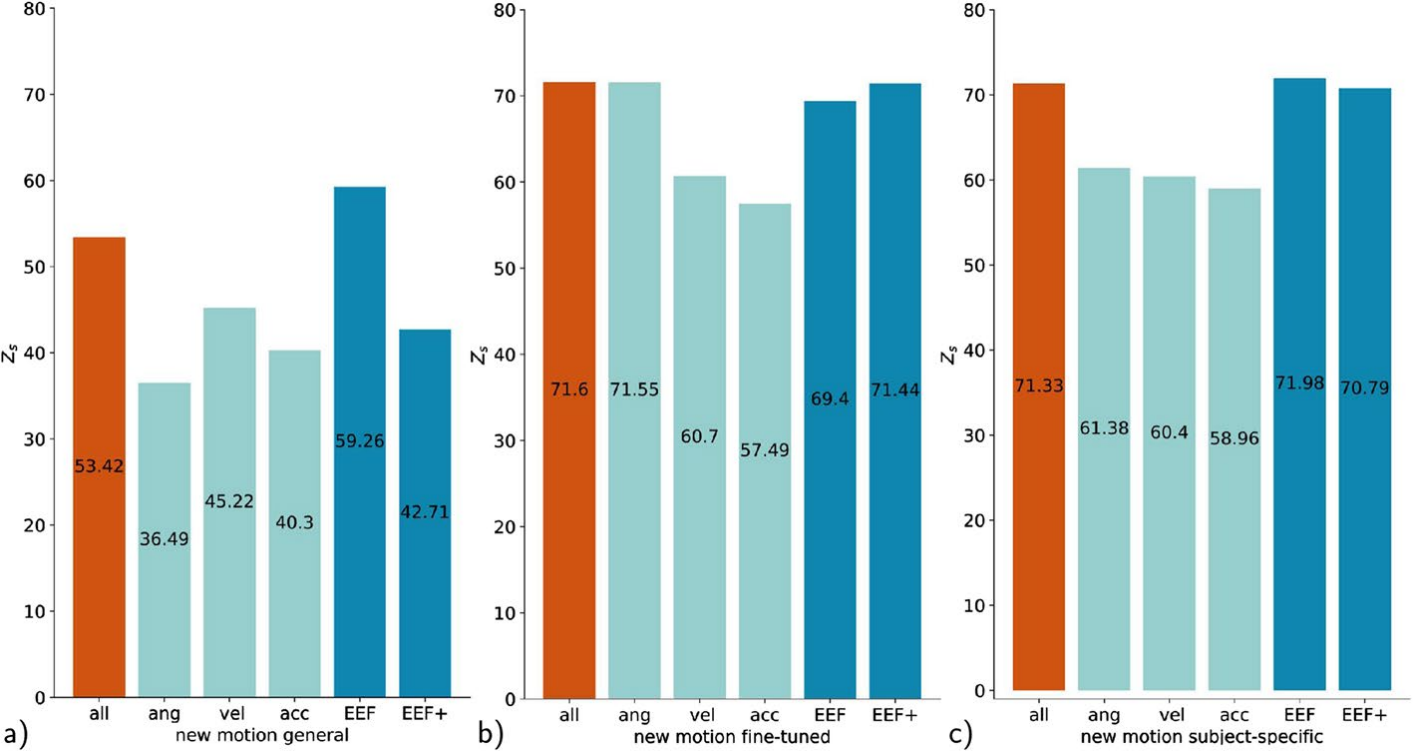
The recurrent neural network (RNN) is separately trained on several parameters: angular position (ang), angular velocity (vel), and angular acceleration (acc) from each joint as well as on the end-effector (EEF) position and orientation of the hand and EEF^+^ with additional velocity and acceleration of the EEF. The RNN is tested on a new motion from the separate subject **a**-**c** and evaluated by zero-line score Z_s_. The results are presented as an average over all channels

Until now, we generated artificial muscle activity for known and new motions based on the chosen input parameter angular position, angular velocity, and angular acceleration for each joint (shoulder, elbow, and wrist). However, from an analytical viewpoint, the latter parameters are redundant since velocity and acceleration can be derived as the first and second derivatives of the position, respectively. Note that with integration on one, the velocity and position can be accumulated from the acceleration, and thus up to numerical precision, only one of the three quantities is needed to describe motion. Therefore, we want to test whether one of these three parameters is sufficient enough as input for a neural network to provide a similar good abstraction for generating known (Table 6) and new muscle activity (Fig. 10 or Table 5). To this end, we train the RNN separately on each input parameter and compare the ability to generate motions to the previous results using all three input quantities simultaneously. Furthermore, instead of testing the motion for each joint as before, we want to consider the position and orientation of the hand as input data, the so-called end effector (EEF) and (EFF+) if the corresponding velocity and acceleration are included as well.

The RNN trained with all input parameters still outperforms all other approaches with a reduced number of input data for a new subject and new motion (Tables 5, 6). The result is particularly evident in the case of the new motion prediction (Fig. 10). For the general and fine-tuned approaches, the accuracy drops from a score of 71.55 for solely using the angular position to 57.49 for the acceleration input. A similar trend can be observed for the subject-specific models. The EEF and EEF^+^ achieve comparable results to the RNN trained with all parameters. However, the subject-specific model slightly outperforms the other models with a score of 71.98.

### Leave-One-Out method: determine variation between subjects

**Fig. 11:**
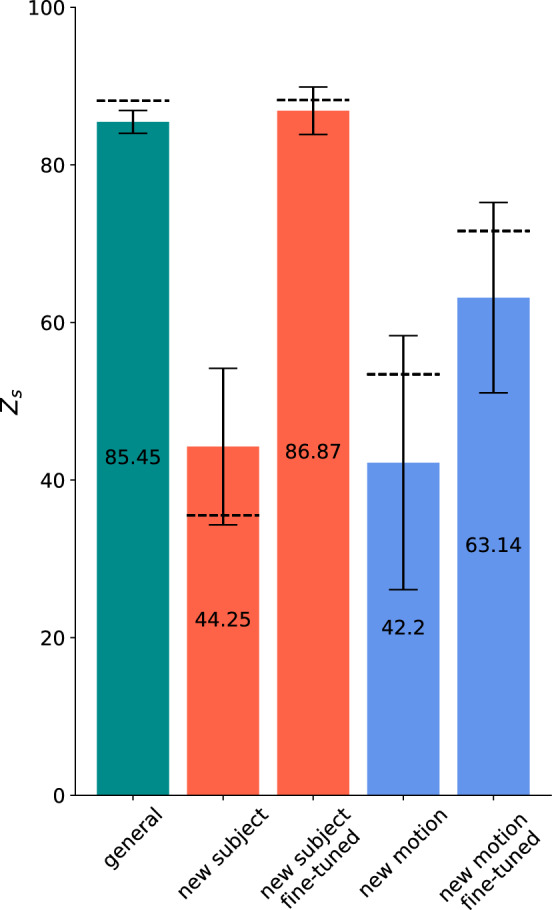
Average performance and standard deviation with the Leave-One-Out method (*n*=4) for all models based on the RNN architecture. The black dashed line represents the result from the original approach (see "Comparison of the network architectures, Performance for new subjects, Generalization property for new motion" sections) with a separated test subject and new motion (3 points in space) used there

In the previous prediction, we choose always to test on the same separate subject and the same new motion, to compare the different approaches with each other. The results will likely vary slightly across individuals as well as the results will differ for new motions. How does subject variation affect our results? Therefore, we want to use the Leave-One-Out (LOO) method to determine the variation between subjects. In the LOO method, one other subject is left out of each run, such that the model can be evaluated based on this subject. After running across all subjects, the results are averaged and displayed with the standard deviation to indicate the variance (Fig. 11). In contrast to "Performance for new subjects" section, where only one subject was tested as an example, the LOO method is used in this Section to make a more general statement about the influence of different subjects. Since the new motion (3 points in space) used prior is not performed by all subjects, two other movements are removed from the pool and used as a test data set for the new motion. These two consist of simple shoulder flexion and a complex relay handover motion. The score of the new motions is average over both new motions. The subsequent calculations have been conducted with the RNN model. As suspected, the variability for the general and fine-tuned model is lower in contrast to the new subject and new motion model. The variability for the new motion decreases again with the fine-tuned model. In addition, the black dashed line indicates the result of the original approach (see "Comparison of the network architectures, Performance for new subjects, Generalization property for new motion" sections). The original model is in most cases within the standard deviation of the LOO method and is thus reproducible. The slightly better result of the initial approach in the general model could be due to a higher number of training data, since two motions as described above were removed for the LOO to be used as a new motion.

### How many repetitions of a motion are required for learning?

**Fig. 12:**
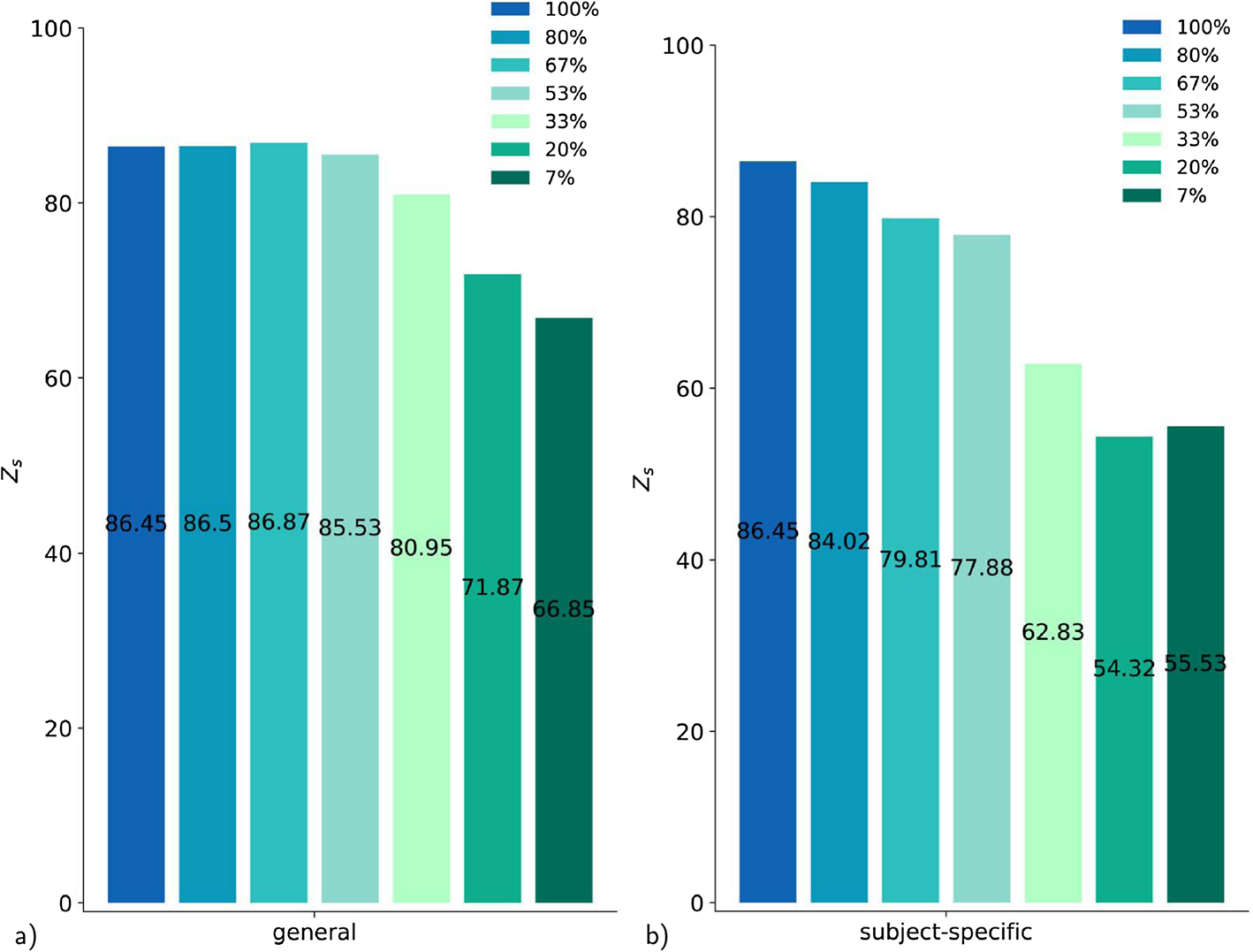
**a** Decaying amount of repetitions (from $100\%\sim 15$ repetitions to $7\%\sim 1$ repetition) predicted by the general model with the RNN architecture. **b** Decaying amount of repetitions (from $100\%\sim 15$ repetitions to $7\%\sim 1$ repetition) predicted by the subject-specific model with the RNN architecture

Furthermore, we want to evaluate how many repetitions of a motion are required to learn the motion. The repetition rate is analyzed by training the general model with different numbers of repetitions ($1\sim 7\%, 3, 5, 8, 10, 12, 15\sim 100\%$ of data) (Fig. 12). Surprisingly, a single repetition already achieves a score of 67 for the general model and 55 for the subject-specific model. The accuracy increases steadily with the quantity of data until about $70\%$ is reached in the general model. Between $100\%$ and $70\%$ no improvement is seen. This tendency does not occur in the new subject or new motion configuration; they are less affected by the number of repetitions. In contrast to the general model, the increasing amount of repetitions lead to a further increase in the subject-specific model score. We empathize that the overall number of data used by the subject-specific model is always lower compared to data of the general model using 4 subjects, and, therefore, has 4 times higher number of repetitions, respectively (see "Discussion" section).

### Subject-specific or general model

**Fig. 13:**
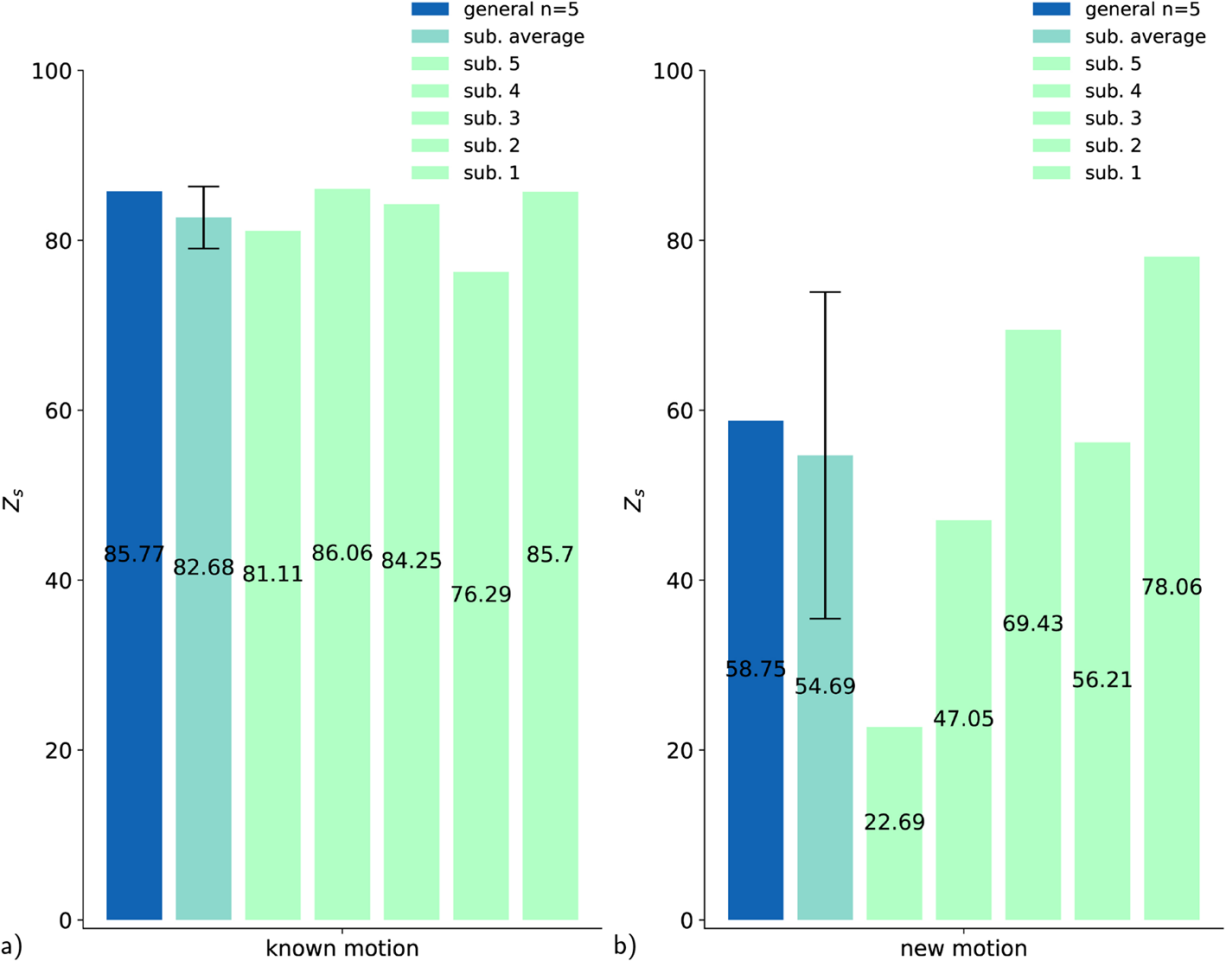
**a** The subject-specific models (1-5) are tested on themself. The added-general model (*n*=5) is tested on all subjects. **b** The subject-specific models (1-5) are tested on themself for the new motions. The added-general model (*n*=5) is tested on all subjects for the new motions

The EMG signal has a high inter-subject variability which makes it hard to predict across subjects. However, in practice, an adaptation to a new subject is often required. The subject-specific model is explicitly trained on this subject during the first application. In contrast, the general model is trained on multiple subjects in advance and benefits from a larger data set to potentially generalize across subjects. The question is whether a subject-specific model is superior to a general model. Therefore, we apply the LOO method introduced above and generate 5 subject-specific models (i.e., one for each subject) and equivalent to that an added-general model trained on all 5 subjects at once. Note that this added-general model should not be mistaken with the general model from all previous "Comparison of the network architectures – How many repetitions of a motion are required for learning?" sections which is always trained on $n=4$ subjects. In the first step, each model is tested on already known motions of the trained subjects. For the subjects-specific model, we train on subject x and test on subject x. Similarly, for the added-general model, which is trained on all subjects ($n=5$) and also tested on all subjects. In the second step, each model is tested on the new motions of the trained subjects in the same way as described above using the shoulder flexion and a relay handover motion. In the first step, the added-general model slightly outperforms the average subject-specific models (Fig. 13). The standard deviation from the subject-specific is remarkable small compared to the second step. For new motions, the score of the subject-specific varies between widely $23-78$ (Fig. 13). The added-general model is again slightly above the average subject-specific model.

## Discussion

The underlying motivation of this work is to demonstrate that artificial muscle activity of known and unknown motion can be generated based on motion parameters such as angular position, acceleration, and velocity of each joint (or the end-effector instead), which are similarly represented in our brains [6–9]. For this purpose, we develop a neural network with a recurrent architecture that is trained in a supervised learning session. Alternative architectures are also elaborated and tested for comparison. Furthermore, we evaluate different training approaches: the general model, the fine-tuned one, and the subject-specific model. All architectures and the majority of the other training approaches produce good artificial muscle activity for previously trained movements. In addition, we also generate artificial muscle activity on new motion, i.e., types of motions that were previously not used to train the network. Naturally, this is a much more challenging task, and trained motions achieve higher similarities than these new motions.

The general setting of comparing different neural network architectures is described in "Comparison of the network architectures" section. The RNN outperforms the other architectures for most muscle activity. In all models, the zero-line score is higher for the second and third channels likely due to an unbalanced amount of data. These two channels represent the shoulder abduction and flexion, which have a higher presentation in the recorded motion, whereas wrist motions are less represented and tend to have lower approximation values (channels seven and eight).

While the general model shows an exceedingly good approximation for all subjects included in the training data, it does not generalize well for new subjects data (Table 3). That is most likely due to the high inter-subject variation of the recorded muscle activity [28]. To minimize these differences primarily caused by varying skin conditions a normalization factor is already calculated and applied for each subject [44]. However, the normalization only accounts for a linear relationship between the subjects; in addition, there are likely to be additional changes in shape caused by individual anatomy and slightly inconsistent placement of the sensors [29–34]. Thus, we must further enhance the model so that it can more accurately predict new subjects’ data as well. The fine-tuned model starts with the weights of the general model, which are further fine-tuned by an additional short training session with the data of the new subject utilizing transfer learning [28, 43]. There are other fine-tuning approaches, however, weight initialization seems to be the most promising for muscle data [45, 46]. Alternatively to the fine-tuned model, a subject-specific model that is purely trained on one subject’s data can also be used. The latter has the same amount of data for training compared to the fine-tuning step of the so called model but has a higher number of epochs. However, it misses the weight initialization step of the fine-tuned model based on the larger dater set of the other subjects. Overall, the fine-tuned model outperforms the average subject-specific model (Fig. 13) both for predicting the subjects data and for new motions. Interestingly, the subject-specific model seems to have a high variance between different subjects for new motions (Fig. 13). This suggests that the subject-specific model does not generalize well in some cases. In comparison, a model trained on multiple subjects seems to be less vulnerable. The influence of the different subjects to a model trained on multiple subjects can be seen in the LOO approach (Fig. 11).

The input parameter validation reveals that a combination of angular position, velocity, and acceleration results in a slightly higher overall accuracy than each parameter on its own, especially by being more robust for new motions (Table 5). This is consistent with findings in motor cortical activity during reaching where besides the movement direction also less dominant correlations as velocity and acceleration are represented [8, 9]. From an analytical point of view, the redundancy of input parameters, for example, velocity as the derivative of position and the integral of acceleration, should not increase the accuracy of the overall model. In contrast to biological systems, and especially the neuronal system, a high degree of redundancy is present. The artificial neural networks inspired by these also exhibit an increase in performance, stability, and faster convergence due to the application of redundancy [47, 48]. The EEF^+^ as an alternative input parameter reaches similar high scores for the fine-tuned model as the RNN with angular position, velocity, and acceleration of each joint. Note that the variation in the EEF may be lower than the variation of the overall sum of all joints in the arm. That could be explained by the fact that the arm has seven degrees of freedom, whereas only six degrees are sufficient to describe the EEF. The former allows choosing slightly different trajectories ending at the same position and orientation of the hand. The addition of velocity and acceleration parameters as further input, which was previously shown beneficial, for the angular position of all joints, causes the EEF^+^ to be able to achieve slightly higher scores for the fine-tuned model.

In an additional step, we verified that our models can generate muscle activity based on new motions. This reveals the true ability to abstract the relationship between motion parameters and muscle activity. As expected, the accuracy for predicting new motions is lower than for the trained motions with a zero-line score of 72 and *r*^2^=0.71 compared to a zero-line score of 88 and r^2^=0.86 for known motions. However, the artificial signal still follows the trend of the original signal well (Fig. 9). Our results are in line with previous works as [19, 26] (see "Background" section) using similar approaches but different task designs and achieving an average *r*^2^ of 0.40 and 0.66 for new 3D motions, respectively. The comparatively lower values of *R*^2^ of 0.36 compared to 0.86 for known motions is due to the fact that the shape and timing is well matched (latter indicated by a high *r*^2^ value) but the amplitude tends to undershoot the original signal here (Fig. 9).

We also investigate how much data are needed for the RNN to learn to predict muscle activity based on motion data. The amount of data is given by three factors: the number of subjects, the number of motions, and their repetitions. The evaluation of the results of the LOO method has confirmed that, especially for new subjects, the prediction accuracy depends on the subject itself and is very variable compared to other new subjects (Fig. 11). Thus, it makes little sense to vary the number of subjects between $1-5$ in this framework to see how many subjects are required. The second factor, the influence of the number of motions and especially the exact nature of the motions, is part of further work. The number of repetitions of a movement has a clear effect on the performance of the model. Saturation is already reached with 10 repetitions for the general model using data of 4 subjects so the model does not improve significantly with more repetitions. However, the subject-specific model continues to improve as the number of repetitions increases. This is most likely due to the fact that the general model is based on multiple subjects, and thus even if each movement is repeated only once, the actual repetition rate adds up to the number of subjects, whereas in the subject-specific model there is only one person and thus only one actual repetition of the movement. It is also remarkable that even one repetition achieve a score of 67 for the general model and 54 for the subject-specific model (Fig. 12). This indicates that repetition of the same motion alone is not necessarily crucial for a good generalization of the model.

Most models generate muscle activity using an entire motion sequence and thus work offline. The RNNseq and FNNseq model are based on sub-sequences and are thus suitable for online prediction, however, it leads to a slightly decrease in overall accuracy.

The recorded muscle activity includes an over-representation of the zero line representing an inactive state of the corresponding muscle. Initially, the motion sequences are already cut to eliminate inter-trial pauses. However, due to the different lengths of each single task, the sequence still incorporates some zero line signals to ensure that all sequences have the same length. Further, not all recorded muscles are active in each motion. Most tasks are designed to activate only certain muscle groups such that all the other channels have a resting signal close to zero. Ensuring the same signal length is especially important for the CNN, while the RNN and FNN can cope with different sequence lengths. While the representation of the zero line itself is meaningful, as it represents the non-active state which is crucial for learning, this over-representation of low values leads to inherently smaller values in the MSE metric, which can easily be misinterpreted. As a result, the zero lines are easily predicted by all models, but learning other values becomes more challenging, and the model naturally tends to form lower peak muscle activity values overall. The newly introduced zero-line score differs from the *R*^2^ by accounting for the zero line whereas the *R*^2^ accounts for the mean value of the signal and thus is naturally suited for rating EMG signals accounting for the over-representation of the zero line.

The RNN receives the highest score for all motions used for training with a zero-line score of 88, while new motions reach an average value up to 72 for the zero-line score and a *r*^2^= 0.81. We emphasize that a theoretical 100 cannot be achieved in real-word applications due to the generalization by training the networks with different subjects, as mentioned before. Furthermore, we already described above that it is more difficult to achieve a high zero-line score for data containing many zero signal segments. Note that the MSE achieves very low values of 0.00066 and 0.00131 (Table 2), respectively. Finally, even for a hypothetical perfect test environment in which a subject can perform two exactly identical movements, it is not clear whether the corresponding muscle activities must also be perfectly matched. Consequently, our models achieve a very high degree of similarity, that can also be seen in Fig. 6.

This work is valuable to support EMG-based classifiers for myoelectrical controlled devices which requires additional data to improve further performance and generalization. In addition, a transfer to a functional electrical stimulation (FES) protocol is conceivable to support the movement of paralyzed limbs. Many studies have already shown that FES has a high impact for rehabilitation of stroke patients [49–52]. The approach involves approximating the relationship between muscle activity and force and then transferring this to stimulation patterns. Our model, is founded on a comprehensive data set that encompasses various motion types and many repetitions, see "How many repetitions of a motion are required for learning?" section. Featuring data from five healthy subjects, excels at predicting muscle activity similar to the original subjects (see "Leave One Out method: determine variation between subjects") but may have limited accuracy for individuals in a rehabilitation setting. For this, recruiting a more diverse group of subjects will supposedly improve the accuracy and generalizability of the model and expand our understanding of muscle activity generation. This is particularly important given the high inter-subject variation in muscle activity and the need in rehabilitation context to also account for age-related and pathological differences. For further work, we will also test our model for the vice versa approach predicting the motion parameters we used in this work as input values from EMG signals to gain more insight into the complex relation between motion and muscle activity. Furthermore, it will be interesting to combine this model with a biomechanical model and compare the expected outcome.

## Conclusions

This work shows that artificial muscle activity of known and unknown motion can be generated based on motion parameters such as angular position, acceleration, and velocity of each joint (or the end-effector instead), which are similarly represented in our brains [6–9]. The dynamic behavior of the motor cortex might be best explained by a recurrent neural network [49–52], which also achieves remarkable results in our case. We obtain outstanding results predicting muscle activity through different subjects. Moreover, the model generalizes over a wide range of motions including new motions. A transfer learning approach was successfully used to overcome the challenging variations in muscle activity between subjects, resulting in a good adaptation of muscle activity for a new subject. The efficient prediction of muscle activity is relevant for the fundamental understanding of movement control and the rehabilitation process of neuromuscular diseases with myoelectric prostheses using functional electrical stimulation.

## Data Availability

The data sets used and/or analysed during the current study are available from the corresponding author on reasonable request.

## Appendix

See Tables 5 and 6

**Table 5:** Recurrent neural network (RNN) is separately trained on several parameters: angular position (ang), angular velocity (vel), and angular acceleration (acc) from each joint. As well as on the end-effector (EEF) position and orientation of the hand and EEF+ with additional velocity and acceleration. The RNN is tested on a new motion from the separate subjects and evaluated by zero-line score Zs and mean square error MSE

RNN		General	Fine-tuned	Subject-specific
All	*Z*_s_	53.42	**71.6**	71.33
	MSE	0.01286	0.00784	0.00791
Ang	*Z*_s_	36.49	71.55	61.38
	MSE	0.01753	0.00785	0.01066
Vel	*Z*_s_	45.22	60.7	60.4
	MSE	0.01512	0.01085	0.01093
Acc	*Z*_s_	40.3	57.49	58.96
	MSE	0.01648	0.01173	0.01133
EEF	*Z*_s_	59.26	69.4	**71.98**
	MSE	0.01124	0.00845	0.00773
EEF^+^	*Z*_s_	42.71	71.44	70.79
	MSE	0.01581	0.00788	0.00806

The results are presented as an average over all channels, with the bold numbers indicating the highest score

**Table 6:** Recurrent neural network (RNN) is separately trained on several parameters angular position (ang), angular velocity (vel), and angular acceleration (acc) from each joint. As well as on the end-effector (EEF) position and orientation of the hand and EEF+ with additional velocity and acceleration. The RNN is tested on the same test data set of the separate subject and evaluated by the zero-line score Zs and mean square error MSE

RNN		General	Fine-tuned	Subject-specific
All	*Z*_s_	35.52	88.21	85.16
	MSE	0.00747	0.00137	0.00172
Ang	*Z*_s_	28.93	80.16	72.86
	MSE	0.01923	0.00823	0.0023
Vel	*Z*_s_	27.0	84.72	69.24
	MSE	0.00846	0.00177	0.00356
Acc	*Z*_s_	5.66	84.08	77.18
	MSE	0.01093	0.00184	0.00264
EEF	*Z*_s_	39.81	87.57	83.56
	MSE	0.00697	0.00144	0.0019
EEF^+^	*Z*_s_	30.41	**89.48**	86.37
	MSE	0.00806	0.00122	0.00158

The results are presented as an average over all channels, with the bold number indicating the highest score

## Abbreviations

EMG: Electromyography
EEF: End-effector
FES: Functional electrical stimulation
MSE: Mean square error
RMSE: Root mean square error
NRMSE: Normalized root mean square error
*r*: Correlation coefficient
*R*$^{2}$: Coefficient of determination
VAF: Variance accounted for
*Z*$_{\textrm{s}}$: zero-line score
FNN: Feedforward neural network
RNN: Recurrent neural network
LSTM: Long short-term memory
CNN: Convolutional neural network
